# Parental Perspectives of a Wearable Activity Tracker for Children Younger Than 13 Years: Acceptability and Usability Study

**DOI:** 10.2196/13858

**Published:** 2019-11-04

**Authors:** Kelly A Mackintosh, Stephanie E Chappel, Jo Salmon, Anna Timperio, Kylie Ball, Helen Brown, Susie Macfarlane, Nicola D Ridgers

**Affiliations:** 1 Applied Sports Science, Technology, Exercise and Medicine Research Centre Swansea University Swansea United Kingdom; 2 Institute for Physical Activity and Nutrition Deakin University Burwood Australia; 3 Learning Futures Deakin University Burwood Australia

**Keywords:** mobile applications, physical activity, child, monitoring, ambulatory, wearable electronic devices

## Abstract

**Background:**

There is increasing availability of, and interest in, wearable activity trackers for children younger than 13 years. However, little is known about how children and parents use these activity trackers or perceive their acceptability.

**Objective:**

This study primarily aimed to ascertain parental perspectives on the acceptability and usability of wearables designed to monitor children’s physical activity levels. Secondary aims were to (1) identify practical considerations for future use in physical activity interventions and promotion initiatives; (2) determine use of different features and functions incorporated into the accompanying app; and (3) identify parents’ awareness of their child’s current physical activity levels.

**Methods:**

In total, 36 children (18 boys and 18 girls) aged 7-12 years were asked to wear a wrist-worn activity tracker (KidFit) for 4 consecutive weeks and to use the accompanying app with parental assistance and guidance. Each week, one parent from each family (n=25; 21 mothers and 4 fathers) completed a Web-based survey to record their child’s activity tracker use, app interaction, and overall experiences. At the end of the 4-week period, a subsample of 10 parents (all mothers) participated in face-to-face interviews exploring perceptions of the acceptability and usability of wearable activity trackers and accompanying apps. Quantitative and qualitative data were analyzed descriptively and thematically, respectively. Thematic data are presented using pen profiles, which were constructed from verbatim transcripts.

**Results:**

Parents reported that they and their children typically found the associated app easy to use for activity tracking, though only step or distance information was generally accessed and some difficulties interpreting the data were reported. Children were frustrated with not being able to access real-time feedback, as the features and functions were only available through the app, which was typically accessed by, or in the presence of, parents. Parents identified that children wanted additional functions including a visual display to track and self-monitor activity, access to the app for goal setting, and the option of undertaking challenges against schools or significant others. Other barriers to the use of wearable activity trackers included discomfort of wearing the monitor because of the design and the inability to wear for water- or contact-based sports.

**Conclusions:**

Most parents reported that the wearable activity tracker was easy for their child or children to use and a useful tool for tracking their children’s daily activity. However, several barriers were identified, which may impact sustained use over time; both the functionality and wearability of the activity tracker should therefore be considered. Overall, wearable activity trackers for children have the potential to be integrated into targeted physical activity promotion initiatives.

## Introduction

### Background

Physical inactivity is identified as the fourth leading cause of death globally [1], with regular physical activity associated with numerous physiological and psychosocial health benefits in youth. Physical activity during childhood plays a critical role in reducing cardiometabolic risk factors, which are associated with long-term health [2]. Government guidelines across the world (eg, Australia, the United Kingdom, and the United States of America) recommend that children engage in at least 60 min of moderate- to vigorous-intensity physical activity (MVPA) every day [3-5]. Given that most children globally fail to meet these recommended levels [6-8], effective and sustainable interventions to increase children’s physical activity levels are needed.

Children’s lack of understanding regarding the amount and intensity of physical activity they do across the day is a frequently cited barrier to their overall physical activity levels [9,10]. This is further evidenced by research supporting the overestimation of self-reported data [11,12], suggesting that youth (children and adolescents) are not able to accurately self-monitor. Given that self-monitoring has been identified as a useful behavior change technique for enhancing awareness of, and potentially stimulating change in, physical activity levels [13], wearable activity trackers could play an important role in improving awareness of children’s physical activity levels among parents and their children.

Over the past decade, there has been significant growth and interest in commercially available wearable activity trackers (eg, Fitbit, Garmin, and Xiaomi), becoming the number 1 worldwide fitness trend in 2017 [14]. Although research has examined the validity and reliability of such wearable devices for measuring key outcomes such as steps, distance traveled, active minutes, and energy expenditure, their potential impact as tools for promoting physical activity remains poorly understood [15,16]. Nonetheless, wearable activity trackers have shown good validity for measuring physical activity in preschool children [17,18], children [19], and adolescents [20], as well as promising acceptability for adolescents [21], adults [22], and older adults [23]; however, little research has been conducted with children younger than 13 years of age. This may be attributed to restricted data storage and access imposed by manufacturers in accordance with the American Children’s Online Data Protection Act. Despite physical activity promotion interventions using wearable activity trackers for a range of populations [24-27], few have targeted youth [28] given that commercially available wearable activity trackers specifically designed for children (eg, KidFit, Fitbit Ace, and Garmin Vivofit Jr 2) have only more recently become available.

Mobile health (mHealth) apps are a promising approach for physical activity promotion, leveraging the ubiquity and pervasive nature of smart devices. When coupled with wearable activity trackers, these self-monitoring systems [29] can provide real-time feedback, as well as other behavior change techniques [30], such as goal setting, peer comparison, social support, and nudges [31]. Masteller et al [32] identified 36 well-established behavior change techniques across a range of features and functions within 3 commercially available youth-oriented activity trackers and accompanying websites [16,29]. However, Riley et al [33] advocated that the capability of wearable activity trackers to enhance active behaviors relies on the device’s ability to engage users to encourage and support positive behavior change in the first place. Owing to data restriction regulations for children younger than 13 years, parents are not only the gatekeepers of children’s physical activity but also gatekeepers to mHealth apps. Therefore, parental perceptions are imperative. Understanding the users’ experience, in this case parents, is essential to provide recommendations for future programs seeking to use activity trackers with children.

To date, research conducted using wearable activity trackers has typically used multiple devices simultaneously, prescribed app/website access and minimum durations, or used child-based questionnaires in isolation [32,34]. Moreover, the implications associated with the storage of children’s data may limit app interaction, and thereby in-depth real-time feedback, an integral component of wearables [16,28], through the requirement to sync via parental/guardian smart devices. Further research is therefore required in free-living settings to understand an individual’s natural use and interaction with such wearable activity trackers.

### Objectives

The aim of this study was to ascertain parental perceptions on the acceptability and usability of wearable activity trackers designed to monitor children’s physical activity levels (<13 years). Secondary aims were to (1) identify practical considerations for future use in physical activity interventions and promotion initiatives; (2) determine and understand the use of different features and functions incorporated into the accompanying app; and (3) identify parents’ awareness of their child’s current physical activity levels.

## Methods

### Participants and Settings

Participants were recruited through an email advertisement circulated to staff and students working at or attending an Australian university. Parents with children aged 7-12 years who did not currently own or had not previously used the KidFit (a commercially available activity tracker) were eligible to participate in the study. In total, 37 families expressed interest in the study, with 25 parents (4 fathers and 21 mothers) providing written informed consent for themselves and their children (n=36; 18 boys and 18 girls) to participate in the study (25/37; 68% response rate). In total, 11 families had 2 children participate. Participation involved children wearing the KidFit activity tracker for 4 consecutive weeks, and parents completing a weekly Web-based survey. Following completion of the fourth week, a subsample of parents (n=10) provided consent to participate in a one-on-one interview. As children were not directly involved in data collection, no written assent for participation was required. Ethical approval was provided by the Deakin University Human Ethics Advisory Group (Health).

#### Wearable Activity Tracker

This study investigated the acceptability and usability of wearable activity trackers specifically designed for children. Acceptability was defined as the perceived usefulness of the device for tracking activity behaviors, although usability was defined as the perceived ease of use of the device [21]. The KidFit (X-Doria International, Santa Monica, CA, USA; AUD $70 per device) was selected as it was smaller and targeted a broader age range than the only other commercially available wearable activity trackers specifically designed for children younger than 13 years of age at the time of the study. However, it is important to highlight that the aim of the study was to assess the acceptability and usability of wearable activity trackers, rather than device-specific information, because of the rapid technological turnover. Nonetheless, the KidFit (Multimedia Appendix 1) is a splash-proof, rechargeable activity tracker (3.5 × 2.2 × 1.0 cm^3^) worn on the wrist using a snap band that collects physical activity and sleep data. No feedback is provided to the wearer about their activity levels; this information is only accessible via the accompanying app, which is free to download from the App Store or Google Play Store. Data are manually synced with the app via Bluetooth when pressing the button on the front of the device. Information provided in the app includes the number of steps, distance traveled (based on steps taken), a KidFit score (based on activity levels), and sleep time, which can be tracked over time (days/weeks) and facilitates goal setting. Challenges are also provided in the app (eg, hit your daily goal 3 times in a row). Similar to other wearable activity trackers for children, the KidFit requires charging approximately every 5 days. It is compliant with the American Children’s Online Privacy Protection Act.

#### Protocol

Each child was provided with a KidFit and asked to wear it for 4 consecutive weeks. Data collection took place between September and December 2015 (Spring-Summer). As participants had not previously used the KidFit, a research assistant met with each parent to help them set the device up for their child. This process involved downloading the KidFit app on to the parents’ smartphone or tablet, showing them how their child should wear the activity tracker, and familiarizing them with basic functions of the activity tracker and app. These included how to charge the KidFit, how to sync it with the app, what the different flashing lights on the KidFit meant, and how to navigate the app. A user manual that was developed by the research team was also provided to parents, which included troubleshooting information on these functions. This manual was based on a quick start manual that was provided with the KidFit and additional information available over Web at the time of the study. No instructions were provided on how to customize daily goals or how to interpret the information generated by the app. Parents were asked to sync their children’s data each day to ensure no data were lost. Parents were not provided any specific directions regarding app interaction frequency or duration, engagement with app content (eg challenges), or data interpretation, with their children.

### Measures

#### Survey

Parents were asked to complete a short Web-based survey at the end of each week that their child wore the activity tracker. Where more than 1 child from each family was participating, parents completed 1 survey per child. Items developed for use in this study (see Table 1) assessed the use of the activity tracker and app in the previous week, daily wear compliance, reasons for nonwear or nonuse, perceptions of the wearable activity tracker and app (eg, ease of use, comfort, awareness of activity, and the ability to understand information provided), enjoyment of use, problems experienced using the device and app, and how information was used by the parents and their children (eg, rewarding goals achieved). All quantitative items were rated on a 4-point Likert scale from 0 (strongly disagree) to 3 (strongly agree). Open-ended items asked about the parents’ perspectives (eg, how did you use the information provided?) and their children’s perspectives (eg, what does your child like about the wearable activity tracker?). Demographic data (eg, age and sex of parent and child) were collected in the survey completed at the end of week 1. Parents’ ownership and use of activity trackers was also assessed in week 1.

**Table 1:** Descriptive survey data on wearable activity tracker use and perceptions among parents (parents were asked to complete 1 survey per child).

Activity tracker use^a^		Week 1	Week 2	Week 3	Week 4
Worn for 7 days, n/N (%)		19/35 (54)	14/35 (40)	19/34 (56)	14/31 (45)
Not worn at all, n/N (%)		1/35 (3)	1/35 (3)	2/34 (6)	4/31 (13)
**Acceptability (% agreeing), n/N (%)**					
	My child likes wearing the activity tracker	30/35 (86)	30/35 (86)	27/34 (79)	28/31 (90)
	My child is embarrassed to wear the activity tracker	1/34 (3)	7/35 (20)	5/34 (15)	6/31 (19)
	The activity tracker has made me think about how I can help my child to be more active	16/34 (47)	17/35 (49)	11/34 (32)	15/31 (48)
	My child is more active than normal *because* they are wearing the activity tracker	16/34 (46)	14/35 (40)	10/34 (29)	7/31 (23)
**Usability (% agreeing), n/N (%)**					
	The activity tracker is easy to use	30/34 (88)	29/35 (83)	25/34 (74)	24/31 (77)
	My child finds the activity tracker uncomfortable to wear	17/34 (50)	20/35 (57)	19/34 (56)	15/31 (48)
	I (or my child) experienced problems in the last week using or wearing the activity tracker	27/35 (77)	20/35 (57)	19/34 (56)	10/31 (32)
	My child had trouble remembering to put on their activity tracker	9/34 (26)	13/35 (37)	13/34 (38)	13/31 (42)
	The activity tracker activity score reflects how active my child is	26/34 (76)	25/35 (71)	23/34 (68)	22/31 (71)
	The activity tracker data are easy to understand	27/34 (79)	30/35 (86)	27/34 (79)	25/31 (81)

^a^The N values differ due to some questions not being completed in the survey.

#### Interviews

The semistructured interviews explored parents’ views about the acceptability and usability of the wearable activity tracker and accompanying app as well as the potential use of commercially available wearable activity trackers as a tool for promoting physical activity in real-world settings. Example questions included:

What things did you (and/or your child) like about the KidFit?

Did you experience any issues using the KidFit?

Can you explain what features you/your child used and why?

Did the KidFit increase your child’s activity and/or your awareness of their activity levels?

How could such devices be used within physical activity programs?

Interviews were conducted by the same research assistant and continued until data saturation was reached. In total, 10 interviews (all mothers) were conducted and digitally recorded. Interviews (mean duration 19.5 [SD 4.4] min) were then transcribed verbatim, producing 81 pages (Times New Roman, size 12) of raw transcription data for analysis.

### Data Analyses

#### Survey Data

All quantitative survey data were collapsed to form binary percentage agreement data. Specifically, *strongly agree* and *agree*, and *disagree* and *strongly disagree* categories were merged to those who agreed and disagreed with each of the statements, respectively. The percentage of parents who agreed was then calculated.

#### Qualitative Data

After reading and undergoing familiarization with the transcripts, 2 researchers (KAM and SEC), independent of the project team and each other, thematically analyzed [35] the transcripts and developed an initial list of codes in NVivo 12 (QSR, Southport, the United Kingdom). Consistent with recommended approaches [36], these authors then discussed their independent coding, as well as any discrepancies and deviant cases, until a consensus was reached. This led to a small number of new codes being developed and existing codes being deleted or refined. Once the transcripts had been fully coded, data under specific codes were retrieved and overarching or central themes identified. Pen profiles—an increasingly used technique to present analysis outcomes via diagrams of composite key emergent themes [37]—were then constructed, with example verbatim quotations provided to further illustrate the theme. To provide an indication of the prevalence of the themes, the number of parents who mentioned a specific theme is also presented [37].

A third author (NDR) with expertise in qualitative analyses [38] then analyzed the data in reverse from the pen profiles back to the transcripts, critically questioning the presented thematic analyses [39]. This process allowed authors to offer alternative interpretations and interrogate the data until a consensus was reached, assuring credibility [37]. The adopted inductive analytic approach enabled within- and between-transcript comparisons, which ensured findings stayed grounded in these data. Overall, methodological rigor (ie, dependability, credibility, and transferability) was demonstrated through the comparison of pen profiles with verbatim transcriptions, verbatim citations, and the bidirectional triangular consensus process, and the likelihood of bias was minimized through the independent coding of transcripts and exploration of deviant cases. To provide a detailed analysis of parental perceptions, both quantitative descriptive data from the surveys and qualitative data from the follow-up interviews are presented.

## Results

### Survey Data

Daily wear compliance and activity tracker perceptions are presented in Table 1. Although the percentage of children wearing the activity trackers for 7 days across the 4-week period fluctuated, there was little variation in the reasons provided for nonwear across the weeks. Reasons included forgetting to wear the activity tracker, needing to charge it or it having a flat battery, or being asked to remove it for sports. Other reasons provided were device malfunction, being ill, losing the device, or staying somewhere other than their primary home. Although 1 child chose not to wear the wearable activity tracker at all beyond the initial set up, another chose not to wear it toward the end of the study because of losing interest as a consequence of the perceived inaccuracy of the device to capture their activity. Parents reported that the reasons some children did not wear the activity tracker at all included the following: *not being into it*; too bulky and restrictive for sports; it malfunctioning; *hating it*; or having lost it. The percentage of parents reporting that their child found the activity tracker embarrassing to wear increased from 2.9% (1/35) in week 1 to 17.1%-20.0% (5/34 to 7/34) across the rest of the weeks, but parents did not report that this affected wear-time.

Although the percentage of parents stating that their child liked wearing the activity tracker decreased during the 4-week monitoring period (see Table 1), the reasons for their child either liking or disliking the device remained consistent. Parents perceived that their child liked wearing the activity tracker because it facilitated goal setting, was colorful, tracked their physical activity, was *fun* and *cool*, enabled peer comparison and competition, and they liked the snap band. Conversely, those whose children did not like the activity tracker said this was because of the device lacking functions and prohibiting water-based activities, as well as being too bulky, *not cool enough*, uncomfortable/sore, and/or a burden.

Parents generally reported that the activity tracker and associated data were easy to use and understand. There was little change over time in how much parents perceived the trackers made them more aware of, or wanted to increase, their child’s physical activity levels. Although parents initially perceived that the activity tracker increased their child’s physical activity levels, the percentage progressively decreased from week 1 to week 4. Approximately half of the parents reported that their children found the device uncomfortable over the duration of the study.

Across the 4 weeks, the reporting of problems associated with the activity tracker decreased. The key problems cited included the poor battery life, not being able to sync the data, and perceived poor tracking of their child’s physical activity data. Parents generally accessed the app with their child, though this decreased from 88.6% (31/35) at week 1 to 60.0% (18/30) following week 4. During the first week, most parents accessed the app with their children at least once per day (16/35; 45.7%), though (9/35; 25.7%) accessed it at least twice per day. App access was mainly to track activity (17/35; 48.6%) and sync data (17/35; 48.6%), with very few children and parents reporting accessing sleep data and setting goals. Over the 4 weeks of the study, there was a shift in the way parents accessed activity data or synced the activity tracker. Whilst the majority used the app with their child (22/34; 64.7%) at the start, the majority tended to access data on their own, without their child (15/28; 53.8%), at the end of this period. The only other times parents accessed the app without their child was to try to resolve problems, such as syncing.

### Qualitative Data

**Figure 1:**
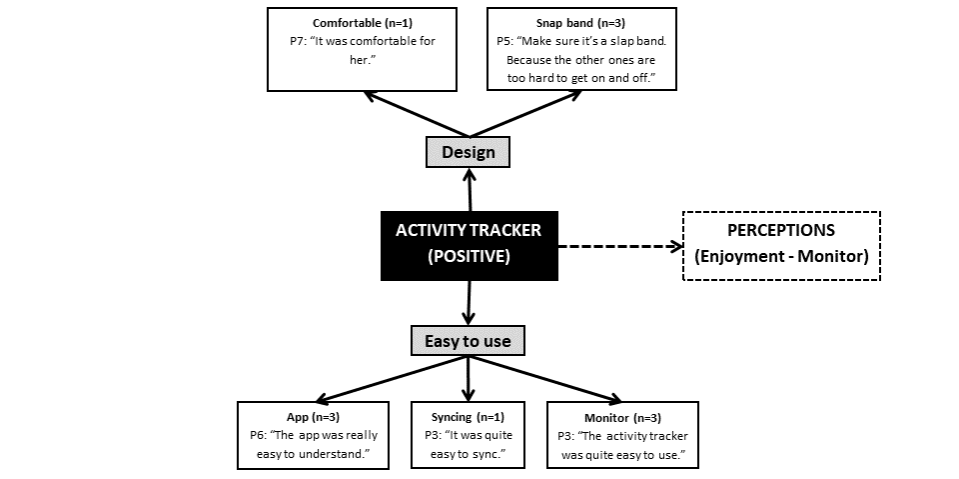
Positive perceptions of the wearable activity tracker. The dashed line indicates link made between different themes noted by the researchers from the points discussed, rather than directly mentioned by the parents (P).

**Figure 2:**
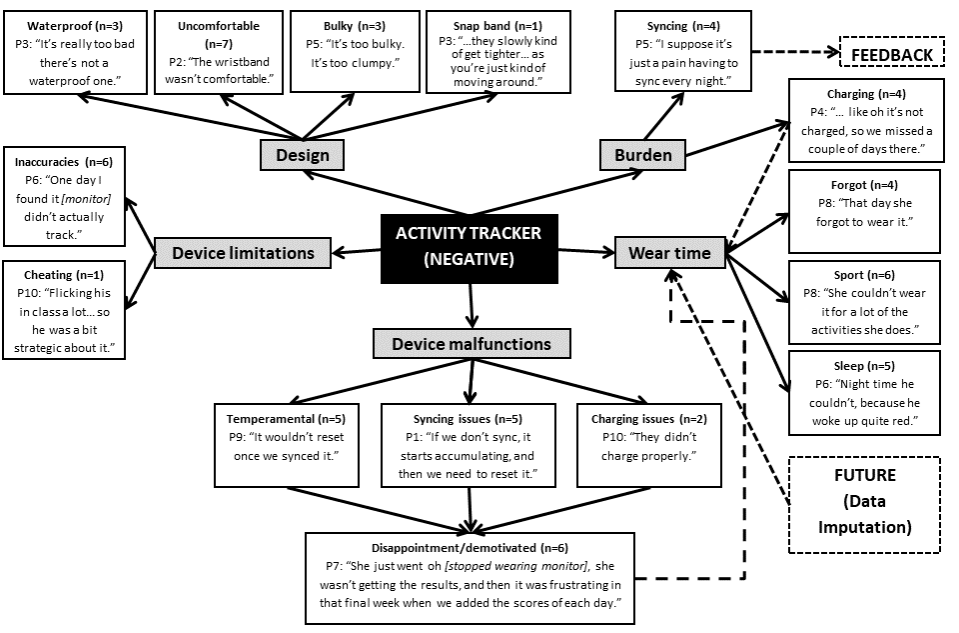
Negative perceptions of the wearable activity tracker. The dashed line indicates link made between different themes noted by the researchers from the points discussed, rather than directly mentioned by the parents (P).

**Figure 3:**
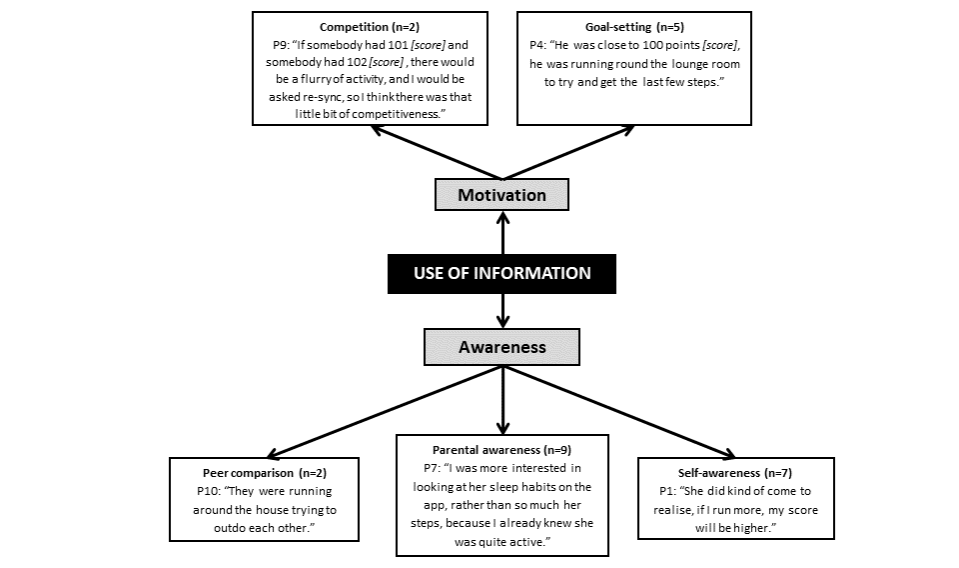
Use of information collected by the wearable activity tracker. P: parent.

**Figure 4:**
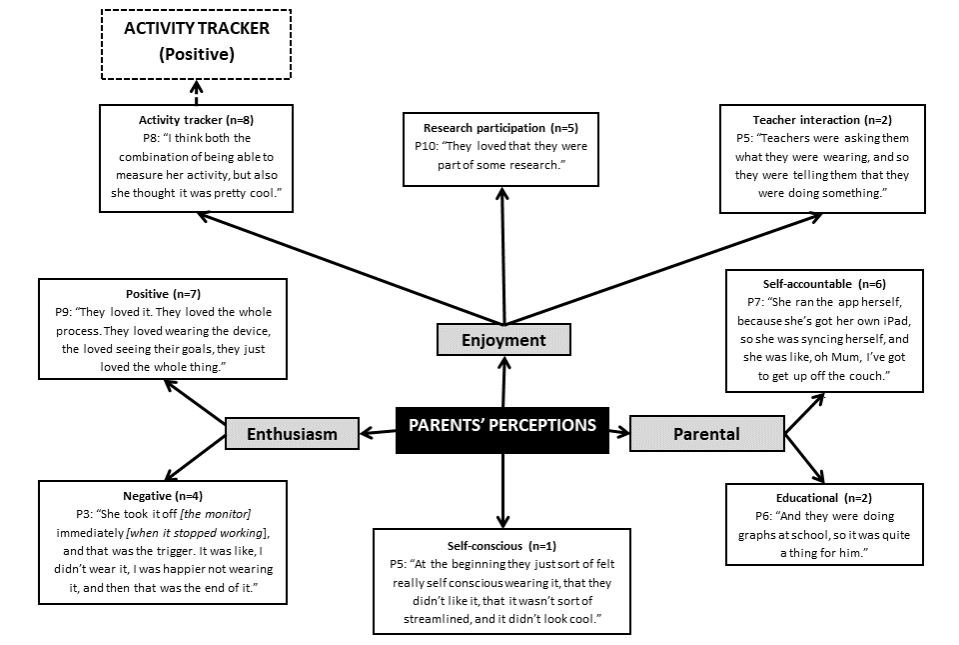
Parents’ (P) perceptions on children’s use of the wearable activity tracker. The dashed line indicates link made between different themes noted by the researchers from the points discussed, rather than directly mentioned by the parents.

**Figure 5:**
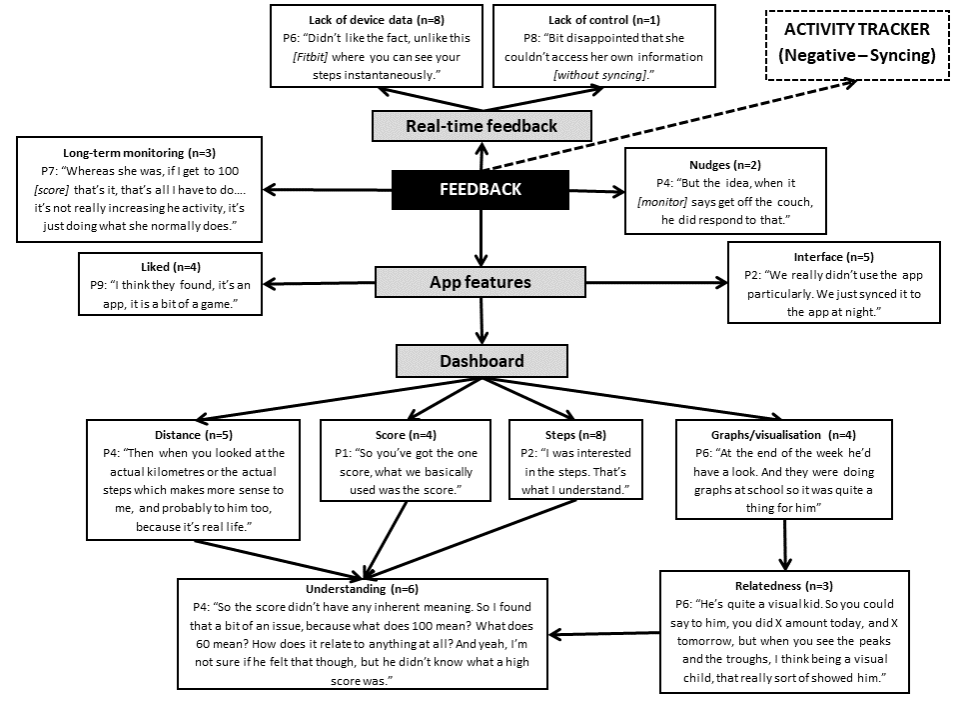
Parents’ perspectives on feedback provided by the wearable activity tracker and how this was used by them and their children. The dashed line indicates link made between different themes noted by the researchers from the points discussed, rather than directly mentioned by the parents (P).

**Figure 6:**
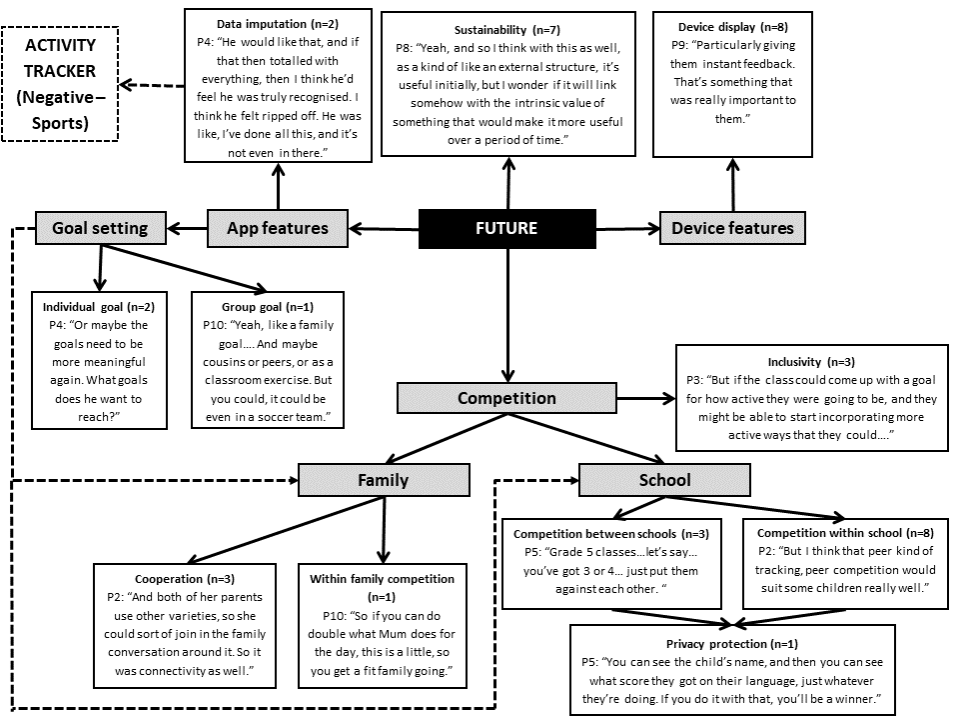
Parents’ considerations and recommendations for using wearable activity trackers in the future. The dashed line indicates link made between different themes was noted by the researchers from the points discussed, rather than directly mentioned by the parents (P).

Data were themed and are presented in 6 pen profiles (Figures 1-6), focusing on the positive and negative aspects of the wearable activity tracker, use of information, perceptions, feedback, and future considerations.

### Wearable Activity Tracker (Positive and Negative)

The activity tracker itself was viewed as both a facilitator (Figure 1) and barrier (Figure 2) to use. Parents reported that the key enablers for their child to wear the activity tracker were the design and ease of use, yet barriers included the burden (ie, syncing and charging the device) and wearability, and its design, limitations and malfunctions. Although some reported the snap band grew tighter across the duration of wear, others highlighted that the band was easy to wear and that their child preferred it. The band was more frequently described as uncomfortable (n=7; 70%) than comfortable (n=1; 10%) among this subsample of parents, with the majority of reasons surrounding the nature of the wristband being tight, hot, and sweaty:

So it slowly would get tighter and tighter, and especially when she’s out running around, it would get really hot, and kind of sweaty underneath the band, because they don’t breathe either.

Parent 3

Furthermore, several parents reported that their children found the activity tracker *bulky* on the wrist. The snap band fastener (ie, not secure), comfort, and lack of device functionality (ie, no digital time display or real-time feedback) were frequently cited as barriers to wearability, as well as to potential long-term use. Several other factors impacted on the use and wearability of the activity tracker, including forgetting to wear it (4/10; 40%) following removal for sport, sleeping or charging, and being unable to wear it while sleeping (5/10; 50%) because of it being painful and waking up with marks. Parents commonly described their child’s frustration that the trackers were not waterproof and had to be removed for certain sports, such as swimming, basketball, and ballet. However, 1 parent described how their child moved the snap band to their ankle to allow their activity still to be captured:

So then she put it on her ankle, and put her sock over the top so that she could still wear it.

Parent 7

The activity tracker itself was identified as easy to use by 30% (3/10) of parents and with others mentioning the ease of syncing (n=1; 10%) and using the app (n=3; 30%). However, several inaccuracies and malfunctions were noted in the sample.

Most parents reported that the device occasionally did not track their child’s activity (n=6; 60%) or reset to a new day resulting in data accumulation and false results (n=5; 50%). There were also issues with syncing (n=5; 50%) and charging (n=2; 20%). These factors collectively led to the children feeling disappointed and demotivated to continue to wear the activity tracker:

Well then she just didn’t want to wear it, or she was kind of like, oh it’s a silly thing.

Parent 8

Parents reported that they had difficulties syncing the activity tracker and the app (n=5; 50%), and that it was a burden to do every night (n=4; 40%):

...with the syncing, it did take me a while to understand first of all how to sync.

Parent 1

In line with this, parents also found that charging the activity tracker was a burden (n=4; 40%), thereby impacting their child’s ability to wear it:

I would sync it, and then I’d put it on sleep mode, if I remembered. Or I’d have to take it off to charge the battery.

Parent 5

### Use of Information

Parents reported that children predominantly used the data provided by the app for motivation to be more physically active or aware of their physical activity levels (see Figure 3). There was consensus among parents that the data provided information on, or verification of, their child’s physical activity levels. Parents (7/10; 70%) mentioned that the information provided within the app also enhanced their child’s awareness of their own physical activity levels, with 2 parents specifically highlighting peer comparison and enhanced motivation, through either competition (n=2; 20%) or goal setting (n=5; 50%).

### Parents’ Perceptions

Most children enjoyed and were enthusiastic about the activity tracker (see Figure 4). The majority of the enjoyment stemmed from the tracker itself being *cool* to wear. However, 1 parent reported that their children initially felt self-conscious wearing the tracker because of its size and bright color. Most parents found that the activity tracker had a positive effect on their child’s enthusiasm for being active. However, several parents (n=4; 40%) identified that the wearable tracker could result in a lack of enthusiasm:

She was uncomfortable wearing it [the wearable activity tracker]. It wasn’t even providing her with immediate feedback to sort of provide her with intrinsic motivation...[she was like] wow this is annoying, I don’t like it, so I’m turning it off.

Parent 3

When asked about their own perceptions, most parents (6/10; 60%) liked that the activity tracker made their children more accountable regarding their own activity and took responsibility for syncing and charging it. Furthermore, 2 parents reported that the use of the tracker and app linked into their children’s current curriculum:

...they were doing graphs at school, so it was quite a thing for him.

Parent 6

### Feedback

The feedback provided through the app was important to the parents and their children (see Figure 5). A common theme was their children’s desire to be provided with real-time feedback to gain further information about their physical activity levels (8/10; 80%). For example, some parents reported that their children expressed a desire to have feedback through a visual display on the wearable activity tracker, rather than just the app, to facilitate continuous monitoring. Although 2 parents enjoyed the nudges the activity tracker provided when *they were being lazy, and [to] get off the couch* (Parent 10), several others perceived the lack of feedback did not, and was unlikely to, help increase their child’s physical activity levels in the long term. Indeed, the ability to access the app only through the parents’ smartphone or tablet was commonly cited as an inhibiting factor to receiving feedback, particularly on weekdays. Conversely, weekend data access was much easier:

On weekends we might check it 3 or 4 times. Because they’d want to see what they’d scored.

Parent 10

However, 1 child had their own smart device, which allowed her to have more control over increasing their activity levels:

...really she ran the app herself, because she’s got her own iPad, so she was syncing herself, and she was like, oh Mum, I’ve got to get up off the couch.

Parent 7

Although some parents really liked and enjoyed the app (4/10; 40%), others found it was acceptable but not of interest for themselves or their children. The most commonly accessed app features included the score, steps, and distance. Parents also used the graphs as a visualization aid to explore how the output from the activity tracker related to their children’s activity across the day:

...obviously it was sitting in the classroom, so you could see, and then when she went out for lunch, so wetalked about that’s when it [physical activity] spiked up.

Parent 7

However, the use of these features differed across all the parents. Some parents demonstrated a lack of understanding regarding various app components, such as the score and its link to total steps and distance covered, the interpretation of the graphs, and, how to meet and set goals. Moreover, some parents did not access the various features available to them. Specifically, 1 parent felt that it was not appropriate to share the app data with their children:

because it then becomes a competition

Parent 5

### Future Considerations

A number of future considerations and suggestions were raised by the parents (see Figure 6). These include the app and tracker features, the use of competition, and whether children younger than 13 years would continue to wear the tracker in the long run. There was a feeling that the app needed to allow children to enter in times when they did physical activity that the wearable could not detect, similar to adult devices. In addition, they felt that some improvements could be made to the goal-setting function to allow more meaningful individual (n=2; 20%) and group (n=1; 10%) goals between family, friends, and teams. Parents conveyed that their child also felt that the activity tracker itself could be enhanced through adding other functions such as heart rate and a time and date display.

One parent suggested the addition of kinetic charging to eliminate the inconvenience of plugging in the activity tracker to recharge. Indeed, parents’ thought that children would capitalize on the opportunity future activity trackers’ could offer, to not only provide real-time feedback on their daily goal but also to challenge themselves or others, either competitively or cooperatively. This was evident from the suggestion of 2 key environments: school and family. From a family perspective, parents suggested connecting family members through the wearable devices, which could also facilitate healthy competition between family members. Indeed, most parents proposed developing a more competitive nature, particularly within school (8/10; 80%) whereby students know what their peers are doing. However, the issue of privacy protection if children’s names are being shown was also raised (see Figure 6). This inclusive group-based competition, regardless of environment, was thought to be a method to increase the inclusiveness of wearing activity trackers beyond just those children who are active:

It would be great to have a competition between the classes, rather than amongst each individual kid, because then they’re helping each other along.

Parent 7

The long-term wear compliance and engagement of an activity tracker was highlighted as an important consideration for future use because of differences between individual children’s motivation. Indeed, some children wanted to purchase their own wearable tracker, whereas there was a novelty effect for others:

I said, oh would you want to wear them, like would you want one of your own? They said no, we’ve kind of used it now.

Parent 10

## Discussion

### Principal Findings

Although self-monitoring systems have the potential to be used to promote physical activity levels in children, few studies have evaluated the acceptability and usability of wearable activity trackers in this age group. The main aim of this study was to examine parents’ perceptions regarding the acceptability and usability of a wearable activity tracker (KidFit) for children over a 4-week period. Approximately half of parents reported that children wore the activity tracker every day and found it easy to use for activity tracking. However, parents noted that their children’s main frustration was not being able to access real-time feedback, with features and functions only accessible through smart devices typically owned by their parents. Indeed, although newer generations of commercially available wearable activity trackers for children have overcome some of the barriers reported in this study, especially the real-time feedback, it is important to consider critical recommendations for all targeted wearable activity trackers as a potential intervention tool, rather than specific to the KidFit per se.

### Comparisons With Prior Work

Although there is a lack of research examining the usability and acceptability of wearable activity trackers in youth [28], research conducted in young children [34], adolescents [21], and adults [22] has indicated that such devices are often viewed favorably. In agreement with previous research, the ease of use of the wearable activity tracker for children was identified as an important factor [21,24,40-44], with parents highlighting that the accompanying app, on the whole, was easy to navigate. Despite this, many visual app features were not accessed, particularly in relation to tracking sleep. This is contrary to Ridgers et al [21], where despite emphasizing a desire to access sleep data, adolescents felt difficulties and discomfort associated with wearing the activity tracker prevented this. Promoting good sleep habits is becoming increasingly important given the introduction of 24-hour movement guidelines in some countries [45], though there are concerns regarding the accuracy of wearables for tracking sleep [46]. The lack of interest in sleep data in this study could be because of the lack of comfort, which led to 50% of the children not wearing the devices overnight. Newer generation wearable trackers should therefore primarily focus on comfort and 24-hour *wearability*, identified as 1 of the 9 key factors that companies should consider when designing activity trackers [47]. Future research should seek to monitor the time children wear the activity trackers daily.

Of interest, the number of perceived issues with the activity tracker reported by the parents substantially decreased over the 4-week period (77.1%-28.6%). Although this could be attributed to parents identifying problems and knowing how to overcome them, this finding may also be explained by the reduction in children wearing the activity trackers over time (ie, those who had problems simply stopped wearing them). This suggests that future research seeking to use wearable activity trackers in children should integrate a familiarization component for key features and functions to ensure ability to access and understand feedback, which includes problem-solving monitoring issues, to try to ensure that that the tracker is used as intended [21]. More specifically, consideration needs to be given to parents, who may require training in the wearable activity tracker and app use [24], and children, where personalization of the device and app in relation to daily goals could be more important [19]. However, ceased wear could also be a result of children understanding what a sufficiently active day encompasses and, therefore, more accurately interpreting their own behaviors, negating wearable use.

Wearable activity trackers have been increasingly used in physical activity and sedentary behavior interventions, using self-monitoring systems that utilize theory-driven behavior change techniques [16,32]. Although several functions and features are incorporated into the KitFit app, such as a daily score and step counts, as strategies to target key behavior change techniques, these are limited compared with the wearables available to adolescents and adults [16] and the new-generation models for children. Nonetheless, 36 well-established behavior change techniques were identified in a recent content analysis across a range of features and functions within 3 commercially available youth-oriented activity trackers [32]. However, contrary to similar research in adolescents [21], parents in this study reported that they and their children used few features and functions, and therefore, the integration of more behavior change techniques may not be beneficial to the child when accessed through a parental app. This could be explored in evaluations for more recently available wearables for children that incorporate more behavior change techniques.

Those children who did use the associated wearable activity tracker app with their parents mainly used it for goal setting, self-monitoring, and individualized behavioral feedback. These behavior change techniques not only are integral to wearable activity trackers [12] but also are the most important components to influence behavior [13,43]. Despite the relatively short study duration, some children started to implement goal-setting strategies, with qualitative data revealing that most parents interviewed felt that the feedback enhanced their awareness of their children’s current physical activity levels. Parent’s also noted such feedback also increased their children’s awareness of their own activity levels. It could be argued that this increased awareness via the feedback provided was the most important outcome, enabling some children to understand the need to enhance, and others maintain, their activity behaviors [48]. Indeed, research has suggested that the development and implementation of self-regulation is a key objective for utilizing wearable activity trackers [49]. However, the accuracy issues inherent to wearable activity trackers raise concerns about the interpretation of associated data, particularly if the output is not directly relevant to government guidelines (ie, minutes of MVPA).

Irrespective of the different self-monitoring systems used across studies, there were numerous similarities in their acceptability by young children [32] and adolescents [21]. Only the tangible activity data (ie, step or distance), on the whole, was accessed in this study, with the KidFit score rarely being used because of a lack of understanding of how it related to actual activity levels. Newer generation activity trackers, such as the Fitbit Ace, overcome this issue by providing time spent being active in accord with government guidelines. Parents emphasized that children wanted to visually track and self-monitor their activity. Indeed, Masteller et al [32] reported that children liked creating and changing the avatars associated with 2 child-specific wearable activity trackers, as a form of visual feedback. Therefore, *how* the activity tracker itself (eg, visual display) and associated app are designed may be fundamental for long-term use. As such, further research is warranted to explore how these constructs are presented on wearable activity trackers and associated feedback mechanisms, and *whether* and indeed *how* they need to change with long-term use.

Congruent with the previous research, the manner in which the wearable activity tracker fastened around the wrist was the biggest issue [21]. However, Müller et al [34] found that the Garmin Vivofit, which has now evolved from a stretchy bracelet to a watch strap, was a feasible wearable bracelet for assessment in children aged 4-10 years, though it is important to acknowledge that the devices were only worn for 7 days and removed for sleep. In this study, discomfort, coupled with device malfunction or maintenance (ie, syncing and charging), often led to frustration and consequently poor wear-time compliance. Congruent with the studies by Müller et al [34] and Shih et al [50], in children and adults, respectively, aesthetic and practical concerns, such as the activity tracker being *too bright* or *bulky*, suggest that the appealing design of the device is a crucial factor for long-term use [47].

Parents cited peer comparison, relating to awareness or competition, as a desired motivational component. During interviews, only 2 parents reported their children to use the feedback for peer comparison or competition, though this is unknown for the broader sample and could be because of the lack of siblings included in the subsample study, thereby limiting such interaction. Nonetheless, in accord with the previous research [32], group features, such as those incorporated into newer wearable activity trackers, may encourage more regular use by children. These findings are similar to those of Masteller et al [32] who found that 75% of children aged 6-11 years would wear the activity trackers more if their peers were wearing them, though it was the social aspects of the associated activity tracker websites that children liked more than the activity tracking features per se [32]. This concurs with the previous research in adolescents, whereby a wearable activity tracker was combined with social media facilitating social comparison and support [26,44,51]. Given the influence of peers on youth physical activity [52], future research should consider how to integrate and promote peer comparison into wearable activity tracker interventions, while simultaneously enhancing autonomy and minimizing peer pressure, or indeed dropout [53,54].

There are numerous strengths associated with this study. Specifically, this is the first to determine the usability and acceptability of wearable activity trackers in children, from a parents’ perspective, using a mixed-methods approach. Moreover, the survey and qualitative data were consistent, with the latter reaching data saturation. Although short in duration, this study was an ecologically valid acceptability and feasibility study, likely precluding the impact of a novelty effect. However, several limitations are noteworthy of discussion. The recruitment strategy (ie, university staff circular) may have resulted in a nonrepresentative sample incorporating participants from a higher socioeconomic background. However, contrary to findings among adults, evidence of socioeconomic gradients in children’s physical activity is equivocal [55], and the generalizability of our findings across socioeconomic strata is unknown. Nonetheless, the recruitment strategy may have resulted in a sample with higher physical activity levels, which may explain the decreased interest across the study duration. Future research should target families from wider socioeconomic backgrounds and lower physical activity levels. It should be noted that although this was only 1 of 2 activity trackers marketed at children at the time, a number of parents owned activity trackers, which may have influenced perceptions of the acceptability and usability of the wearable activity tracker. Moreover, the KidFit is no longer in production and has since been superseded by newer devices, including the release of the Fitbit Ace, which has addressed issues such as real-time feedback and integrated familial and peer competition. Finally, although this study provides insights into children and parents’ initial experiences, via parental report, of using a wearable activity tracker for children, it is not known *whether* or *how* this would impact longer-term use.

### Conclusions

Taken together, parents reported that the wearable activity tracker was easy to use and a useful tool for tracking their children’s daily activity. However, compliance in wearing the activity tracker was modest and declined across the study. Although parents acknowledged that the wearable activity tracker was acceptable for use in children, there were numerous barriers highlighted, which may preclude long-term wear and monitoring. Further research is needed to determine whether these findings are reflective of generic wrist-worn commercially available wearable activity trackers. Although wearable activity trackers for children hold promise as relatively inexpensive, scalable methods for the delivery of evidence-based behavior change techniques, it is important to address potential barriers and behavior change efficacy. These findings provide insights into how children and parents engage with wearable activity trackers designed for use by younger children (<13 years), which in turn may help to identify how to integrate wearable activity trackers into physical activity interventions or initiatives for children and what factors may need to be addressed to try to facilitate longer-term sustainability.

## Appendix

Multimedia Appendix 1 KidFit monitor.

## Abbreviations

mHealth: mobile health
MVPA: moderate- to vigorous-intensity physical activity
NHMRC: National Health and Medical Research Council
