# Supplementary figures and images for: Parental Perspectives of a Wearable Activity Tracker for Children Younger Than 13 Years: Acceptability and Usability Study
Source: JMIR Mhealth Uhealth. 2019 Nov 4;7(11):e13858. doi: 10.2196/13858 (PMC6861996; doi:10.2196/13858)

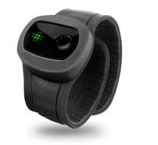

Supplement: Multimedia Appendix 1 [file mhealth_v7i11e13858_app1.png]
